# Pregnancy and Birth Outcomes among Women with Idiopathic Thrombocytopenic Purpura

**DOI:** 10.1155/2016/8297407

**Published:** 2016-03-22

**Authors:** Diego F. Wyszynski, Wendy J. Carman, Alan B. Cantor, John M. Graham, Liza H. Kunz, Anne M. Slavotinek, Russell S. Kirby, John Seeger

**Affiliations:** ^1^Pregistry, Los Angeles, CA 90045, USA; ^2^Epidemiology, OptumInsight, Waltham, MA 02451, USA; ^3^Children's Hospital Boston, Boston, MA 02115, USA; ^4^Medical Genetics Institute, Cedars Sinai Medical Center, Los Angeles, CA 90048, USA; ^5^Palo Alto Medical Foundation, Mountain View, CA 94040, USA; ^6^Department of Pediatrics, Division of Genetics, University of California, San Francisco, San Francisco, CA 94115, USA; ^7^College of Public Health, University of South Florida, Tampa, FL 33612, USA; ^8^Department of Medicine, Division of Pharmacoeconomics and Pharmacoepidemiology, Brigham & Women's Hospital, Harvard Medical School, Boston, MA 02120, USA

## Abstract

*Objective*. To examine pregnancy and birth outcomes among women with idiopathic thrombocytopenic purpura (ITP) or chronic ITP (cITP) diagnosed before or during pregnancy.* Methods*. A linkage of mothers and babies within a large US health insurance database that combines enrollment data, pharmacy claims, and medical claims was carried out to identify pregnancies in women with ITP or cITP. Outcomes included preterm birth, elective and spontaneous loss, and major congenital anomalies.* Results*. Results suggest that women diagnosed with ITP or cITP prior to their estimated date of conception may be at higher risk for stillbirth, fetal loss, and premature delivery. Among 446 pregnancies in women with ITP, 346 resulted in live births. Women with cITP experienced more adverse outcomes than those with a pregnancy-related diagnosis of ITP. Although 7.8% of all live births had major congenital anomalies, the majority were isolated heart defects. Among deliveries in women with cITP, 15.2% of live births were preterm.* Conclusions*. The results of this study provide further evidence that cause and duration of maternal ITP are important determinants of the outcomes of pregnancy.

## 1. Introduction

Immune (idiopathic) thrombocytopenic purpura (ITP) is an autoimmune disorder characterized by persistent thrombocytopenia due to antibody binding to platelet antigen(s) and causing their premature destruction by the reticuloendothelial system, particularly in the spleen. The American Society for Hematology guidelines [1] define ITP as “isolated thrombocytopenia with no clinically apparent associated conditions or other causes of thrombocytopenia.” Therefore, ITP is a condition generally diagnosed by exclusion of the numerous other causes of thrombocytopenia, such as infections, medications, hematological malignancies, disseminated intravascular coagulation, and other autoimmune conditions. In ITP, persistent thrombocytopenia is associated with an otherwise normal full blood count [2]. Chronic ITP is defined as ITP persisting for more than 6 months [3].

It is estimated that thrombocytopenia (defined as a platelet count less than 150 × 10^9^/L) occurs in approximately 7% of pregnant women, with 74% of those with low platelet counts having incidental thrombocytopenia of pregnancy that can be managed routinely and in which the platelet count remains more than 70 × 10^9^/L [4]. Additional causes of thrombocytopenia include complications of hypertensive disorders in pregnancy (21%) and immunological disorders of pregnancy, including ITP, systemic lupus erythematosus, and other secondary causes of immune thrombocytopenia (4%) [4]. ITP occurs in 1 to 2 of every 1000 pregnancies, which in the United States represents about 3000 to 6000 cases of ITP in pregnancy per year [5].

Several studies have examined pregnancy outcomes for women with ITP and found that most pregnancies were uneventful, with successful outcomes for both mothers and children [6–8]. The majority of studies used data from single medical centers [6, 9, 10], by employing retrospective analyses of medical charts [10–15]. Most studies have not differentiated between incidental thrombocytopenia, ITP, and cITP in women who have low platelet counts during pregnancy, and, to our knowledge, there are few published reports regarding the outcomes of pregnant women with cITP.

The primary objective of this study was to examine the pregnancy and birth outcomes of women with ITP and cITP identified from medical claims and abstracted medical records from a large health plan in the United States. Our hypothesis was that there would be no significant difference in adverse pregnancy outcomes, including congenital anomalies, between the two groups of women (ITP versus cITP). The method of data ascertainment enabled the inclusion of data from multiple medical centers, thus increasing the size of the patient cohort and extending the study to a broader population.

## 2. Materials and Methods 

### 2.1. Study Design

We studied pregnancy and birth outcomes among women with ITP or cITP over 16 years of age from 1994 to 2009 inclusive. The data for this study were derived from a large US health insurance database based on eligibility, pharmacy claims, and medical claims data.

### 2.2. Data Source

This study utilized STORK (Systematic Tracking of Real Kids) to identify pregnancies and link the health experience of mothers with that of their infants within an administrative claims database in order to study the effects of maternal exposures and health conditions on pregnancy outcomes [16, 17]. Further details about STORK are provided in Supplementary Material available online at http://dx.doi.org/10.1155/2016/8297407. After obtaining institutional review board (IRB) approval and waiver of patient authorization from the affiliated privacy board, medical records were accessed and reviewed to ascertain covariate information and to confirm outcomes.

### 2.3. Study Population

The study population consisted of women with at least one diagnostic claim for ITP (ICD-9 code 287.31 or 287.3 if used before 01/12/2005) and at least one diagnostic or procedure code related to pregnancy (live birth, spontaneous abortion, stillbirth, or therapeutic abortion) in the database between 1995 and 2009. To be eligible for the study, women with at least one claim for ITP had to fulfill the following criteria: have complete medical and pharmacy benefit coverage, have at least 280 days of continuous enrollment before the pregnancy outcome date, and have a 9-month baseline period prior to the estimated date of conception. If a woman had more than one pregnancy during the study period, analyses included the first pregnancy after the first ITP claim, ignoring any subsequent pregnancies. If a woman's first ITP claim was later than the end date of her last pregnancy, she was not eligible for this study. The database was then searched for infants associated with the deliveries and linked them to the mother's claims. Linkage between mother's and newborn's claims could not be performed if the infant was enrolled in another health insurance plan, as may happen if the partner has a different health insurer.

### 2.4. Definition of cITP and Timing of Diagnosis Relative to Pregnancy

Patients were classified as having cITP if they met at least one of these requirements: two or more claims for ITP separated by at least 6 months; a claim for ITP and treatment occurring at least 6 months later with one or more of these medications: corticosteroids, anti-D antibody, rituximab, danazol, colchicine, and dapsone; and/or a claim for splenectomy after the first diagnostic ITP claim. Indicators for the timing of the ITP or cITP diagnosis relative to pregnancy were based on the estimated date of conception and the end of pregnancy. Patients were classified as having ITP or cITP before pregnancy if they met the full diagnostic criteria before the estimated date of conception. Patients were classified as having ITP or cITP during pregnancy if they met the full diagnostic criteria before the end of the pregnancy. Women who had an initial claim for ITP prior to delivery or termination of pregnancy but met the full diagnostic criteria for cITP after pregnancy were considered to have ITP during pregnancy, but not cITP, since they did not meet the full criteria until after the pregnancy ended.

### 2.5. Outcomes

The primary outcome of interest was the prevalence of major congenital anomalies (MCAs) at birth (i.e., anomalies that cause significant functional or cosmetic impairment, require surgery, or are life-limiting). Other birth outcomes evaluated included 3 or more minor congenital anomalies, preterm birth (<37 weeks of gestation), low birthweight (<2,500 grams), and measurements consistent with small for gestational age (SGA) status (weight, length, or head circumference below the 10th percentile for sex and gestational age) among infants born to mothers with ITP or cITP. Pregnancy outcomes in this study included live born infant, spontaneous abortion, elective termination, and stillbirth. Potential outcomes were identified by a search for the qualifying diagnosis and procedure codes. If the claims search did not identify a pregnancy outcome, the pregnancy outcome was classified as “unknown.” Claims were reviewed to identify medical providers and facilities to query from them medical records to confirm pregnancy outcomes and to collect additional data on pregnancy characteristics and infant follow-up for the first year of life.

### 2.6. Chart Abstraction

Each patient's pregnancy medical records were abstracted to obtain relevant covariate data from their prenatal and pregnancy outcome history, including estimated date of conception and date and type of pregnancy outcome. For infants with evidence of a congenital anomaly diagnosed within the first 12 months after birth, as identified through ICD-9 diagnosis or procedure codes in the medical claims, the medical record was sought from the physician or hospital where the anomaly was diagnosed. A standardized medical record abstraction form was used to record elements from each mother's chart, including the reported estimated date of conception, type of pregnancy outcome, and other pregnancy characteristics from relevant clinician notes, inpatient records, and/or hospital discharge summaries.

### 2.7. Adjudication of Infant Outcomes

Patient information drawn directly (without abstraction) from deidentified medical records of infants with claims-based congenital anomalies was combined with claims data and reviewed by a dysmorphologist to validate the diagnosis.

### 2.8. Maternal Characteristics and Comorbidities

Following medical record review, all pregnancies were classified according to covariates describing maternal characteristics, pregnancy characteristics, and maternal comorbidities and according to whether they were identified through claims or medical chart abstraction. Maternal characteristics included age and year at conception, geographic region of health plan, and ethnicity. Pregnancy characteristics included use of prenatal vitamins, amniocentesis, chorionic villus sampling, fetal monitoring, ultrasonography, alpha-fetoprotein (AFP) testing, obstetric panel, multiple gestation, high-risk pregnancy supervision, antepartum hemorrhage, abruptio placentae, placenta previa, excessive vomiting, early or threatened labor, late pregnancy, measurements consistent with SGA, disorders relating to short gestation and unspecified low birthweight, and total cost of care for up to 280 days before delivery. Maternal comorbidities such as diabetes, hypertension, and infections, along with substance abuse and receipt of teratogenic drugs during pregnancy, were identified using diagnostic codes and medication claims.

### 2.9. Statistical Analysis

Descriptive statistical analyses were conducted using SAS® (Cary, NC). Frequency analyses were carried out for categorical variables and means and standard deviations were calculated for continuous variables, such as the number of physician visits.

Data were analyzed separately for women who fulfilled the criteria for ITP or cITP prior to pregnancy and those who fulfilled the criteria during pregnancy. For infants with claims of congenital anomalies, data were categorized according to maternal age at conception as follows: 14 years and under, 5-year intervals from 15 years through 49 years, and over 50 years.

## 3. Results

**Figure 1:**
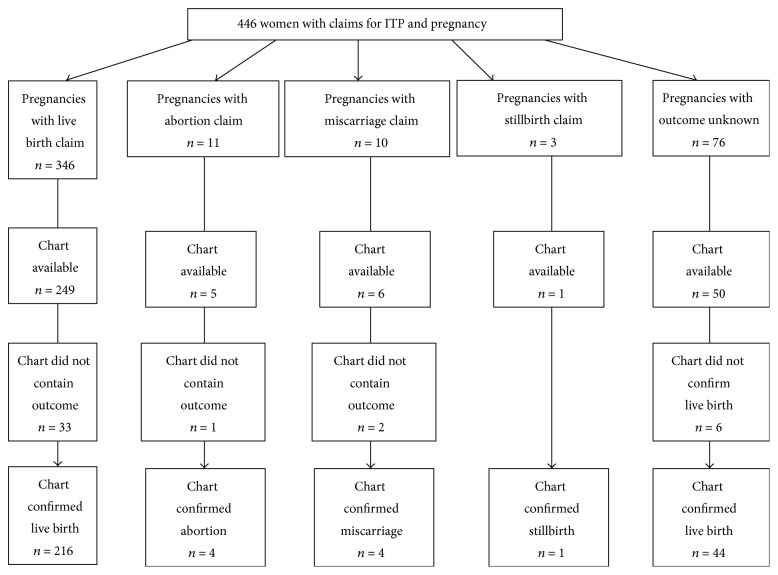
Chart review results of claims for pregnancy outcomes.

We identified 585 women with at least one claim of ITP and claims indicating pregnancy during the study period (January 1, 1994, through December 31, 2009). Of those, 139 did not meet the eligibility criteria and were excluded. The remaining 446 women made up the claims-based ITP cohort for this study. Medical records were sought to provide more detailed information on the patients' pregnancy characteristics not captured in the claims data, and charts for 311 of 446 women (69.7%) were obtained.  Figure 1 provides a schematic view of the claims-based pregnancy outcomes observed in this cohort of 446 women with claims for ITP and the results of chart-based review. Among the 311 women with charts, outcome data were unavailable for 42 of them. Of the remaining, 260 charts had a confirmed live birth and 9 (3.3%) indicated a fetal loss with no further details.

Among the 446 pregnancies in women with a claims-based diagnosis of ITP, the mean age at pregnancy was 30.3 (SD: ±5.3) years. The claims-based diagnosis was available in 432 cases before or during the pregnancy (Table 1). Approximately half of the women reside in the South/Southeast regions of the United States and, of those with ethnicity reported in the enrollment data, approximately 70% are Caucasian. Of the 446 women, 84 (18.8%) were identified as having cITP before or during pregnancy.

Of all 446 pregnancies with claims-based diagnosis of ITP, 346 (77.6%) indicated a live birth (Table 2). When stratified by timing of ITP diagnosis, live births were more frequent among women diagnosed with ITP during pregnancy (290 of 357 or 81.2%) than in pregnant women diagnosed with ITP before pregnancy (56 of 89 or 62.9%). Of those who were diagnosed with ITP during pregnancy, 14/357 (3.9%) were fetal losses compared with 10/89 (11.2%) of those diagnosed with ITP prior to pregnancy. The magnitude and trend of this difference (7.3%) were not found among women with cITP (9/66 or 13.6% of those diagnosed during pregnancy resulting in fetal loss compared to 2/18 or 10.2% among those diagnosed prior to pregnancy).

The prevalence of low birthweight was higher among women with a diagnosis of ITP before pregnancy (10 of 56 or 17.9%) compared to women with a diagnosis of ITP during pregnancy (28 of 290 or 9.7%). The prevalence of low birthweight in women with cITP was similar in both groups, but the sample size was too small to reach a conclusion.

Table 3 presents the prevalence of major congenital anomalies (MCAs) among the 346 infants of mothers with claims for ITP. There were 27 infants (*n* = 27/346; 7.8%) who had a claim for at least one major malformation and charts were available for 17 of them. The most frequent claims were for ostium secundum type atrial septal defects [*n* = 10/346 (2.9%)], hypospadias [*n* = 8/151 (5.3%)], patent ductus arteriosus [*n* = 6/346 (1.7%)], and ventricular septal defect [*n* = 4/346 (1.2%)]. A total of 4 infants (1.2%) had claims for 3 or more major malformations. Of all infants of mothers with claims for ITP that were assumed to have no MCA and whose charts were obtained (*n* = 336), 10 (3.0%) had at least one chart-confirmed major malformation (Table 4). The most frequent of these confirmed MCAs were hypospadias [*n* = 4/336 (1.2%)], ventricular septal defect [VSD, *n* = 3/336 (0.9%)], and patent ductus arteriosus [PDA, *n* = 2 (0.6%)]. Two infants (0.6%) had 3 or more confirmed major malformations.

Among the subgroup of 68 infants of women with claims for cITP, 7/68 (10.3%) had a claim for at least one major malformation. The most frequent claims were for hypospadias [*n* = 4/30 (13.3%)], ostium secundum type atrial septal defects [*n* = 2/68 (2.9%)], and patent ductus arteriosus [*n* = 2/68 (2.9%)]. Two of these 68 infants (2.9%) had claims for 3 or more major malformations. Of all infants of mothers with claims for cITP that were assumed to have no MCA and whose charts were obtained (*n* = 66), 4 (6.1%) had at least one chart-confirmed major anomaly (Table 4). The most frequent of these major congenital anomalies was hypospadias [*n* = 3/66 (4.5%)]. One infant (1.5%) had claims for 3 or more confirmed major malformations. There was no statistically significant difference between the prevalences of malformations in the infants of mothers with ITP compared to those with cITP, regardless of whether the data was claims-based or chart-based (Table 4).

## 4. Discussion

The risks associated with ITP in pregnancy remain controversial [4]. Although severe maternal or neonatal bleeding is rare when pregnant women with ITP are managed by an expert team, a recent questionnaire of women with ITP revealed that 14/50 women were advised to avoid becoming pregnant [18]. Our study is unique because of the availability of data on congenital anomalies in infants born to mothers with ITP and cITP. In addition, the sample size of the population we evaluated is larger than previously published.

The findings of this study are consistent with other published reports. In one prior publication, preterm birth was present in 16/58 (27.6%) infants born to mothers with ITP diagnosed prior to pregnancy and in 15/75 (20%) infants born to mothers with ITP diagnosed during pregnancy, with an overall prevalence of 23.3% [15]. Premature birth affects 5–10% of newborns in most developed countries and approximately 12% of live births in the United States. Therefore, a prevalence of prematurity of 23.3% among pregnancies complicated by ITP represents approximately a 2- to 4-fold increased frequency. In a study by Debouverie et al. [14] of 50 women with cITP who had platelet counts below 150 × 10^9^/L for at least one year, there were no fetal deaths in 62 pregnancies but 9 (14%) were premature, 6 (9%) were small for gestational age, and 2 (3%) demonstrated evidence of hemorrhage. In a group of women treated for severe thrombocytopenia (platelet count < 10 × 10^9^/L) during pregnancy, the average gestational age at delivery was 36 weeks [13]. There was one intrauterine death and the remaining 25 infants were born without complications [13]. Won et al. [11] studied pregnancies among 30 women with ITP or cITP. There were 29 live births from 31 pregnancies, with a mean gestational age of 36.5 weeks (range, 7–43 weeks). There was one missed abortion at 7 weeks and one termination because of intrauterine death at 16 weeks of gestation [11]. A third newborn died because of premature delivery and respiratory failure at 27 weeks. This child was born to a mother who had complications of a bleeding gastric ulcer due to severe thrombocytopenia (platelet count < 20 × 10^9^/L) and who died from acute pulmonary edema following a caesarean section. The remaining 28 newborns had no complications.

The duration of ITP may be important for determining the risk of complications. Thrombocytopenia attributed to aplastic anemia or myelodysplasia was associated with a 53.8% rate of premature birth compared to incidental thrombocytopenia in pregnancy (11.3%) and ITP (16.7%) [12]. Namavar Jahromi et al. [15] contrasted the characteristics of infants born to 57 mothers with ITP diagnosed before pregnancy to 75 women diagnosed with ITP during pregnancy. In the group of mothers with ITP diagnosed prior to pregnancy, there were 2 intrauterine fetal deaths at 224 and 247 days of pregnancy and a higher frequency of infants requiring admission to the neonatal intensive care unit (20/57 or 34.48% versus 12/75 or 16%; *p* = 0.01). Maternal age and platelet count, gestational age at delivery, 5-minute Apgar scores <7, rate of caesarean deliveries, mean neonatal birthweight, and the mean neonatal platelet count did not differ between the two groups. There were three neonates (3/84; 2.3%) with platelet counts < 50 × 10^9^/L that were all born to mothers with ITP diagnosed prior to pregnancy, but there were no severe bleeding complications and no intracranial hemorrhage in the infants. However, maternal ITP refractory to splenectomy has been correlated with a higher risk of intracranial hemorrhage in the infants [19].

Webert et al. [10] conducted a retrospective study of women with ITP in pregnancy, most of whom (83/92) had ITP prior to pregnancy. There were 119 pregnancies and two fetal deaths: one stillbirth at 39 weeks of gestation and a stillbirth at 27 weeks of gestation that had extensive hemorrhage throughout the brain, born to a mother with a 4-year history of severe ITP (postsplenectomy) and platelets counts < 50 × 10^9^/L. There were no reports of fetal malformations and the authors estimated that the fetal loss rate was approximately 1-2%.

No case of hydrocephalus was found in the present study. Hydrocephalus can occur as a rare complication of intracranial hemorrhage in fetuses born to mothers with ITP [20]. Kim and Choi [21] described a neonate with severe thrombocytopenia (platelet count 1 × 10^9^/L), multiple bruises on the face and scalp, widespread petechiae, cleft palate, and moderate to severe hydrocephalus without evidence of intraventricular hemorrhage. Computerized tomography confirmed severe hydrocephalus without significant compression of the brain parenchyma, diffuse ischemia, and encephalomalacia of both cerebral hemispheres.

Ostium secundum, a type of atrial septal defect (ASD), is found in less than 1% of newborns in the general population [22]. In our study, this congenital heart defect occurred approximately three times more frequently among infants born to mothers with ITP. In infants born to mothers with cITP, the incidence of ostium secundum was 2/68 (2.9%) and there were two additional infants with ostium secundum plus other cardiac malformations (Table 3). Ventricular septal defect and patent ductus arteriosus were also noted in other babies born to mothers with cITP (Table 3). However, the numbers in the group of cITP pregnancies were small and the significance of this finding is unclear.

This study provides a claims-based and chart-based evaluation of pregnancy outcomes among women with ITP and cITP in the United States. The wide range inclusion criteria and large source population (more than 1.2 million pregnancies) allowed us to obtain results that reflect pregnancy outcomes broadly among women with ITP and cITP. However, the database has some limitations and valuable information may be missing or misdiagnosed. We aimed to minimize these limitations by performing medical record abstraction. This effort resulted in further data, including additional maternal characteristics and birth outcomes. However, there were a small number of pregnancy cases in women with ITP and cITP with no linkage to births. In addition, data from women with different medical or pharmacy coverage and from those without medical coverage could not be assessed by this study. Anomalies documented in live newborns and those requiring billable medical intervention were assessed in this study. However, minor or major anomalies that may have occurred in stillborn infants and in fetuses of elective or spontaneous abortions were not available for analysis. In addition, clinical validation of the diagnoses of ITP and cITP by a specialist was not feasible in this study. While referral to a maternal-fetal specialist is likely for these patients, the documentation from specialists was generally not included in the primary obstetrical records. Finally, the dataset did not include maternal medical conditions other than ITP, nor medical treatments performed during the pregnancy for ITP or other conditions. Therefore, it was not possible to evaluate their potential effects on the pregnancy and birth outcomes.

## 5. Conclusion

Based on the evaluation of 446 pregnant women with ITP, a diagnosis of ITP or cITP prior to their estimated date of conception may indicate a higher risk for stillbirth or fetal loss, premature delivery, and infants with specific congenital anomalies than an ITP diagnosis during pregnancy. Therefore, the results of this study provide further evidence that the duration of maternal ITP may be an important determinant of the outcomes of pregnancy.

## Supplementary Material

The Supplementary Material provides detailed information about STORK (Systematic Tracking of Real Kids), including the source of data, the process to review the claims profile, and the measure to maintain privacy and confidentiality.

## Figures and Tables

**Table 1:** Demographic characteristics of women with ITP and cITP.

	ITP		cITP	
	(446)		(84)	
	*N*	%	*N*	%
Age at pregnancy				
15–19	14	3.1	4	4.8
20–29	180	40.4	32	38.1
30–39	237	53.1	46	54.8
40+	15	3.4	2	2.8
Race				
African American	18	4.0	3	3.6
Asian	9	2.0	2	2.4
Caucasian	162	36.3	35	41.7
Hispanic	24	5.4	2	2.4
Unknown	213	47.8	36	42.9
Other race	20	4.5	6	4.6
Region				
Midwest	138	30.9	29	34.5
Northeast	55	12.3	10	11.9
South/Southeast	206	46.2	37	44.1
West	47	10.5	8	9.5
Other diseases				
Diabetes mellitus or gestational diabetes	38	8.5	6	7.1
Hypertension or pregnancy-related hypertension	50	11.2	7	8.3

**Table 2:** Pregnancy outcomes among women with ITP and cITP.

Outcomes	All pregnancies among women with ITP		ITP prior to pregnancy		ITP during pregnancy	
	*N*	%	*N*	%	*N*	%
	446	100	89	100	357	100
Live birth	346	77.6	56	62.9	290	81.2
Spontaneous or elective termination or stillbirth	24	5.4	10	11.2	14	3.9
Outcome unknown	76	17.0	23	25.8	53	14.9
Premature delivery^1^	38	8.5	10	11.2	28	7.8
Low birthweight^1^	6	1.4	1	1.1	5	1.4

Outcomes	All pregnancies among women with cITP		cITP prior to pregnancy		cITP during pregnancy	
	*N*	%	*N*	%	*N*	%
	84	100	18	100	66	100

Live birth	57	67.9	11	61.1	46	69.7
Spontaneous or elective termination or stillbirth	11	13.1	2	10.2	9	13.6
Outcome unknown	16	19.0	5	27.8	11	16.7
Premature delivery^1^	8	9.5	2	11.1	6	9.1
Low birthweight^1^	1	1.2	1	5.6	0	0.0

^1^Premature delivery defined as delivery at less than 37 weeks of gestational age; low birthweight defined as birthweight less than 2500.

**Table 3:** Claims-based congenital anomalies among newborns of women with ITP or cITP.

Congenital anomaly	Pregnancies with ITP		Pregnancies with cITP	
	*N*	%	*N*	%
	346	100.0	68	100.0
Any anomaly	27	7.8	7	10.3
Ostium secundum type atrial septal defect	10	2.9	2	2.9
Hypospadias	8	5.3^*∗*^	4	13.3^*∗∗*^
Patent ductus arteriosus	6	1.7	2	2.9
Ventricular septal defect	4	1.2	1	1.5
3 or more anomaly codes	4	1.2	2	2.9
Unspecified congenital cataract	1	0.3	—	—
Bulbus cordis anomalies and anomalies of cardiac septal closure and common truncus	1	0.3	1	1.5
Congenital stenosis of pulmonary valve	1	0.3	—	—
Congenital stenosis of aortic valve	1	0.3	1	1.5
Hypoplastic left heart syndrome	1	0.3	—	—
Congenital anomalies of pulmonary artery	1	0.3	—	—
Congenital tracheoesophageal fistula, esophageal atresia, and stenosis	1	0.3	—	—
Esophageal atresia	1	0.3	—	—
Congenital hypertrophic pyloric stenosis	1	0.3	—	—
Congenital dislocation of hip, bilateral	1	0.3	—	—
Congenital dislocation of one hip with subluxation of the other hip	1	0.3	—	—
Other congenital anomalies of abdominal wall	1	0.3	—	—
Ostium secundum type atrial septal defect + hypoplastic left heart syndrome + patent ductus arteriosus + congenital anomalies of pulmonary artery + esophageal atresia	1	0.3	—	—
Ostium secundum type atrial septal defect + patent ductus arteriosus	2	0.6	—	—
Ostium secundum type atrial septal defect + patent ductus arteriosus + other congenital anomalies of abdominal wall	1	0.3	—	—
Ostium secundum type atrial septal defect + congenital stenosis of aortic valve + patent ductus arteriosus	1	0.3	1	1.5
Ostium secundum type atrial septal defect + ventricular septal defect + patent ductus arteriosus	1	0.3	1	1.5

^*∗*^Denominator: 151 male infants.

^*∗∗*^Denominator: 30 male infants.

**Table 4:** Prevalence of congenital anomalies by ITP and cITP and by source.

Diagnosis	Claims-based	Chart-based
ITP	27/346 = 7.8%	10/336 = 3.0%
cITP	7/68 = 10.3%	4/66 = 6.1%

*P* > 0.05 comparing ITP to cITP.
