# Supplementary material for: Pregnancy and Birth Outcomes among Women with Idiopathic Thrombocytopenic Purpura
Source: J Pregnancy. 2016 Mar 22;2016:8297407. doi: 10.1155/2016/8297407 (PMC4820621; doi:10.1155/2016/8297407)
Supplement: Supplementary file 1 — The Supplementary Material provides detailed information about STORK (Systematic Tracking of Real Kids), including the source of data, the process to review the claims profile, and the measure to maintain privacy and confidentiality. [file 8297407.f1.docx]

**Supplementary Data**

*Data Source*

This study utilized STORK (Systematic Tracking of Real Kids), a process to identify pregnancies and link the health experience of mothers with that of their infants within an administrative claims database in order to study the effects of maternal exposures and health conditions on pregnancy outcomes (16,17). This process, carried out among the covered members of a large health insurer affiliated with Optum provides data for pregnancies resulting in live births, stillbirths, reported spontaneous abortions, and elective terminations. Sourced from Optum’s proprietary research database, containing claims and enrollment data dating back to 1994 and capable of linking patient and physician data to pharmacy and medical claims, medical record data, socioeconomic measures, and clinical laboratory results this linkage adds 80,000 to 100,000 pregnancies each year. Linkage of mothers and infants is carried out using a family identifier along with matching the date of delivery and date of birth. Approximately 30%-40% of identified pregnancies cannot be matched to an infant, either because the pregnancy did not result in a delivery or because the infant was not covered by the same health insurance as the mother. The linked database used in this particular study covered 15 years (1995-2009) and included over 1,200,000 pregnancies, of which over 700,000 infants could be linked to a specific birth mother.

***Health Care and Pharmacy Claims Data***

Health care claims and administrative data were derived from claims submitted by providers and pharmacies to obtain payment for health care services rendered, data to track plan membership for premium billing, and provider data to track participating physicians who have contracts with health plans to provide services. These data included information related to health care costs, resource utilization, quality, and effectiveness. Medical claims or health care encounter data were collected from all available health care sites (inpatient hospital, outpatient hospital, emergency room, physician's office, surgery center, etc.) for virtually all types of services provided, including specialty, preventive and office-based treatments. Pharmacy claims data included drug name, drug strength, fill date, quantity dispensed, and days of supply.

***Claims Profile Review***

Detailed claims profile reviews of pregnancy outcomes among women with ITP or cITP and among their infants with presumed congenital anomalies were performed by clinical consultants to exclude false positives (e.g. transposition of ICD-9-CM digits, coding errors, etc.).

***Privacy and Confidentiality***

Confidentiality of patient records was maintained at all times. All analyses of electronic claims were performed using de-identified data. An internal Disclosure Analysis was performed to verify that Health Insurance Portability and Accountability Act (HIPAA) standards for de-identified data were met. In conformance with requirements of HIPAA, appropriate privacy board and institutional review board approvals were obtained prior to performing chart abstractions.
